# Neurophysiological hyperresponsivity to sensory input in autism spectrum disorders

**DOI:** 10.1186/s11689-016-9162-9

**Published:** 2016-08-08

**Authors:** Yukari Takarae, Savanna R. Sablich, Stormi P. White, John A. Sweeney

**Affiliations:** Center for Autism and Developmental Disabilities, Department of Psychiatry, University of Texas Southwestern, 5323 Harry Hines Blvd., Dallas, TX 75390 USA

**Keywords:** Individual differences, Heterogeneity, Contrast sensitivity, Autism, Sensory hypersensitivity

## Abstract

**Background:**

Atypical sensory processing is a common clinical observation in autism spectrum disorder (ASD). Neural hyperexcitability has been suggested as the cause for sensory hypersensitivity, a frequently reported clinical observation in ASD. We examined visual evoked responses to parametric increases in stimulus contrast in order to model neural responsivity of sensory systems in ASD.

**Methods:**

Thirteen high-functioning individuals with ASD and 12 typically developing (TD) individuals completed a steady-state visual evoked potential study. Stimuli were vertical circular gratings oscillating at 3.76 Hz at varying contrasts (5, 10, 20,…, 90 % contrast, 10 levels). The average spectral power at the stimulus oscillation frequency was calculated for each contrast level.

**Results:**

The magnitude of evoked sensory responses increased at a significantly greater rate and resulted in disproportionately elevated activation with higher contrasts in the ASD group. Approximately 45 % of ASD participants had rates of response increases greater than any TD participant. This alteration was highly associated with parental reports of these participants’ sensory difficulties.

**Conclusions:**

Greater increases in visual responses over contrast manipulation suggest heightened excitability in the sensory cortex in ASD participants. Heightened neural excitability was observed in a substantial portion but not all of the ASD participants. This pattern suggests that individuals with higher excitability may constitute a neurobiologically distinct subgroup requiring individualized treatment interventions.

## Background

Dating back to the earliest characterization of the autism syndrome by Kanner [1], atypical sensory processing has been recognized as a common clinical observation in autism spectrum disorders (ASD) [2–5] and is present throughout the life span [6]. Atypical sensory/perceptual processing has long been believed to have downstream effects on higher-order cognitive processes and social and communication skills in ASD, suggesting a broad relevance of sensory disturbances to core clinical characteristics of ASD [7–12]. While sensory problems are now recognized as a DSM-5 diagnostic criterion for ASD, mechanistic understandings of sensory processing alterations remain notably limited compared to other features of the disorder.

Hypersensitivity to sensory stimuli is one frequently documented symptom in ASD in both parent- and self-reports of sensory experiences [6, 13, 14], and neural hyperexcitability has been suggested to be the cause for such hypersensitivity [15, 16]. Few experimental studies, however, have addressed psychophysical or neural mechanisms related to sensory hypersensitivity. The majority of psychophysical studies have focused on defining perceptual thresholds in ASD, and the thresholds, which determine minimal information required for detecting or discriminating stimuli, are different from reactions to more intense stimuli that are often highlighted in parent- and self-report studies. Few available studies have examined responses to a range of suprathreshold stimuli in ASD, to cover low to high levels of sensory input. Prior work documented greater increases in ASD in perceived loudness of auditory stimuli [17] and increases in blink startle responses to moderately intense visual stimuli [18]. Functional imaging studies have shown elevated activation in visual cortex with suprathreshold visual stimuli [19, 20]. The current study employed a parametric approach to examine electrophysiological responses to suprathreshold visual stimuli across a wide range of visual contrast. The aim was to determine whether there is a propensity for heightened neural excitability in ASD under conditions of increasing visual contrast.

## Methods

### Participants

Participants were 13 high-functioning individuals with ASD and 12 typically developing (TD) individuals that are group-matched on chronological age (ASD 17.46 (SD 5.83), TD 19.75 (SD 6.58) years old, *t* < 1) and performance IQ (ASD 106.00 (SD 17.68) and TD 116.17 (SD 9.62), *t*(23) = 1.76, n.s.) obtained using the Wechsler Abbreviated Scale of Intelligence. All participants in the ASD group met DSM-5 criteria for ASD using the Autism Diagnostic Interview-Revised (ADI-R) [21] and the Autism Diagnostic Observation Schedule-2 (ADOS-2) [22, 23]. The Adolescent/Adult Sensory Profile [24] was completed to report sensory difficulties in everyday situations, and visual and auditory scales were combined to arrive summary scores for each participant. Participants with ASD were excluded if they had an associated infectious, genetic, or metabolic disorder known to cause ASD features such as fragile X or tuberous sclerosis or known clinical history of mood or psychotic disorders. No participants had a history of epilepsy or of taking lithium, antipsychotic, or anticonvulsant medications. One participant was taking antidepressant medication at the time of testing, and none were taking psychostimulants.

TD participants reported no personal history of psychiatric or neurological disorder, no known family history of ASD, and no first-degree relative with a neuropsychiatric disorder considered to have a genetic component. They had no personal history of developmental delay, learning disability, or significant problems in school performance, based on parent/self-report. No participant had a history of neurologic disorder or birth injury. Far acuity of all participants was normal or corrected to at least 20/40. Informed consent and/or assent were obtained from all participants and, when appropriate, from their parents/guardians. The study was approved by the Institutional Review Board of the University of Texas Southwestern where all EEG recoding and clinical assessments were performed.

### Stimuli

**Fig. 1:**
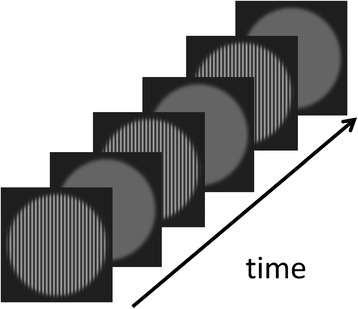
Illustration of the stimulus used in the current study

Stimuli consisted of vertical achromatic sinewave gratings (~2 cycles per deg (cpd)) presented through a circular aperture (13 deg diameter). The sinewave grating alternated with an average luminance-matched plain gray background with an on/off frequency of 3.76 Hz, using a square temporal profile (Fig. 1). The average luminance in all stimulus conditions was kept constant (~32 cd/m^2^). Stimuli were presented for 24.5 cycles (6.5 s) with 1.3–2.3 sec randomly selected intertrial intervals. There were 10 stimulus contrast conditions (5, 10, 20,…, 90 %) randomly assigned to each stimulus presentation, with each contrast condition presented 14 times (total of 364 cycles of stimulus per condition). Both the luminance and contrast of stimuli were confirmed using a luminance meter (Minolta, LS-110, Ramsey, NJ). Stimuli were presented with a small circular fixation point (.24 deg diameter) placed at the center of the grating to aid central fixation, and participants were instructed to keep their eyes on the fixation mark during the task. Frequent breaks were used to reduce fatigue, and the typical task duration including breaks was approximately 20 min. Visual stimuli were presented using an LED monitor (Samsung SA700, Ridgefield Park, NJ, 2 ms response time, 120 Hz) and Presentation software (Neurobehavioral Systems, Berkeley CA). A chin rest was used to maintain a constant viewing distance at 78 cm to the stimuli.

### EEG recording and analysis

EEG recording was performed in a dimly lit, quiet room. Ninety-two equidistant Ag/AgCl active electrodes embedded in a soft fabric cap with external wires (Brain Vision LLC, Morrisville, NC) were used to record EEG. The electrode coverage was slightly larger than the standard 10-10 system to extend below the inion. Electrooculography (EOG) was recorded to monitor blinks and eye movements. The SMI RED500 eye tracking system (SensoMotoric Instruments, Boston, MA) was used to monitor eye position to ensure center fixation during task performance. When participants failed to maintain central fixation, the task was paused and participants were reminded to keep their eyes at the center of the stimulus. Electrode positions were recorded to derive 3D scalp topography for individual participants using a Fastrak digitizer (Polhemus, Colchester, VT) in order to correct for individual differences in cap fit and placement. Impedance was adjusted prior to EEG recording while viewing the impendence map in real-time using the BrainVision actiCHamp System (Brain Vision LLC, Morrisville, NC). Impedances for the active electrodes were kept below 30 kΩ. All physiological signals were amplified and digitized continuously at 1 kHz (DC to 200 Hz) and stored for off-line analysis.

In order to estimate the magnitude of sustained brain response to the stimulus, data recorded during the first four stimulus cycles of oscillating sinewave gratings were excluded to reduce influence of transient brain response to stimulus onset. The remaining data were processed using BESA 6.0 (BESA GmbH, Gräfelfing, Germany). Artifact scoring was performed, using 1024-ms non-overlapping windows, with individualized thresholds because of inter-participant differences in baseline signal amplitude and signal to noise ratios. Both amplitude (ASD ±123.27 (SD 30.64), TD ±117.63 (SD 35.93) μV) and gradient (amplitude difference between two successive samples, ASD 120.94 (SD 35.14), TD 118.22 (SD 33.91) μV) thresholds were applied, and data segments were excluded if any channel exceeded either the amplitude or gradient thresholds. The thresholds did not differ between groups (*t*’s <1) and they were not related to age or IQ (*r*’s = −.40 to −.07, all n.s.). Numbers of data epochs after artifact rejection did not differ between groups: 543.08 segments (SD 59.20) for the ASD group and 560.17 (SD 67.83) for the TD group (*t* < 1). Then, the data were averaged and average referenced. The electrode locations were transformed into the standard 81 locations of the 10-10 system using spline interpolation [25], based on individual participant’s electrode and fiducial locations recorded with the Fastrak digitizer.

Following past studies [26, 27], spectral power of the response at each stimulus contrast level was estimated using Discrete Fourier transformation (DFT) in BESA 6.0 to index the magnitude of neural responses to the visual stimuli. Spectral power at the first and second harmonics of the stimulus frequency was estimated and combined per stimulus contrast, using 1024-ms moving windows with 50 % overlap. A cosine squared function was applied to the data to reduce edge effects during the Fourier transformation. 3D activation maps of spectral response were created in order to identify peaks in the occipital region in individual data. While a single electrode is often used in visual studies to extract information related to peak activation, it has been shown that individual anatomical differences in visual cortex produce slightly different topography for activation during a steady state paradigm [28]. We observed that approximately 16 % of ASD participants had a peak at Oz and 62 % at POz. And, approximately 42 % of TD participants had a peak at Oz and 25 % at POz. Thus, the nearest electrode to the peak location was selected to extract the response magnitude for the individual participant. DFT was also performed for prestimulus periods using −1100 to −76-ms epochs to estimate spectral power magnitude during intertrial periods at the same location, in order to individually scale the response magnitude during the task period. Then, the scaled power magnitude was entered into a group × stimulus contrast ANOVA for statistical analyses. Bootstrapping procedures with 2000 samples were used to determine the significance of statistical effects. If the ASD group were to have an increased neural excitability to sensory input, a greater increase in spectral power of electrocortical response with increasing stimulus contrast would be expected.

## Results

**Fig. 2:**
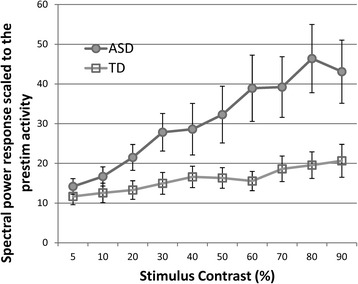
Average spectral power over stimulus contrast manipulation for the ASD and TD groups

Spectral power response as a function of stimulus contrast increased in both groups (Fig. 2), *F* (9, 207) = 12.49, *p* < .001. The response magnitude overall was greater in the ASD group (*F* (1, 23) = 5.71, *p* < .05), and the group difference was progressively greater as stimulus contrast increased (group × contrast interaction, *F* (9, 207) = 4.52, *p* < .05 (with Greenhouse-Geisser correction)).

**Fig. 3:**
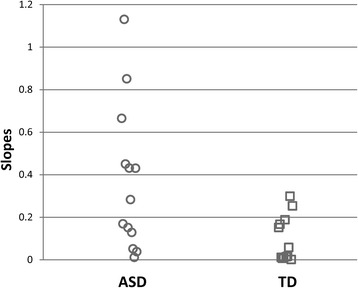
Linear slopes fitted to individual data to quantify rates of response increases over contrast manipulation for each group

To provide a single scalar index of this increased neural excitability in relation to the contrast manipulation, linear functions were used to determine the slope of each participant’s spectral power by stimulus contrast function. As expected based on the group level analysis, individual slopes were overall higher in the ASD group (Fig. 3) (ASD .37 (SD .34), TD .10 (SD .11), *t*(23) = 2.62, *p* < .05, Cohen’s *d* = 1.07). No group differences in individual intercepts were observed (ASD 14.09 (SD: 8.35), TD: 11.50 (SD: 5.58), t < 1).

The examination of individual subject data showed substantial heterogeneity within the ASD group, with some patients showing a marked increase of neural responses and other subjects overlapping with the TD sample. Six out of 13 ASD participants (approximately 45 %) had slopes that were completely outside the range of the TD participants. No ASD individuals had data indicating a reduced neural response relative to the TD participants.

**Fig. 4:**
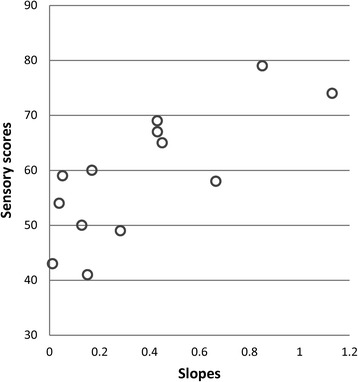
Scatterplot showing that greater individual slopes for response increases are associated with more items endorsed in clinical reports of visual and auditory symptoms in ASD participants

Because of these individual differences, we investigated correlations between individual subjects’ slopes and clinical variables (age, IQ, ADOS and ADI summary scores, sensory ratings) in the ASD group. Neither age nor PIQ correlated with slopes of the contrast to neural response function: *r* = −.15 (age), *r* = −.14 (PIQ), n.s. Individual slopes were only modestly correlated with social affective scores on ADOS (*r* = .44, n.s.), and correlations with other ADOS and ADI summary scores were lower and nonsignificant. However, slopes reflecting neural excitability were significantly positively correlated with ratings of visual and auditory sensory symptoms from the Sensory Profile questionnaire, *r* = .77, *p* = .01 (Fig. 4).

## Discussion

We examined visual neural responses to a wide parametric range of stimulus contrast manipulations in the suprathreshold range. Both ASD and TD groups demonstrated contrast dependency by showing graded, increasing neocortical responses as a function of stimulus contrast. Neural responses, however, increased at a significantly greater rate in the ASD group, indicating that neocortical activation becomes disproportionately greater with more intense stimulus contrast in the ASD group. The correlation of this abnormality with reports of sensory sensitivities in everyday settings was significant, suggesting a clinical relevance to the atypical neurophysiological functioning.

Increased cortical excitability has been proposed as a fundamental neurological characteristic of ASD [15, 16]. This proposal has gained support from genetic and epigenetic studies of ASD that document gamma-aminobutyric acid (GABA) [29–34] and glutamate [35–37] system alterations. Because neocortical sensory systems rely on a set of gain control mechanisms that depend on a precise balance of excitatory and inhibitory tone, dysregulation of the balance affects a wide range of sensory processes with downstream effects on perceptual functions. This is consistent with a recent MR spectroscopy study documenting changes in relationships between cortical GABA concentrations and visual perceptual performance among ASD participants [38].

Generation of inhibitory currents via GABAergic signaling is known to affect levels of regional pyramidal neurons’ readiness to produce action potentials in response to inputs [39, 40]. It has been shown that the rates of neural response to input increase following administrations of glutamate agonists [41] and GABA antagonists [42]. It is noteworthy that our result of greater neural responsivity to increasing sensory input resembles those previously reported in epilepsy patients in a similar paradigm [43, 44] while shared etiology between epilepsy and ASD has been discussed by some authors [45]. Further, our finding parallels observations from a meta-analysis of fMRI studies, which indicates that enhanced activation in visual cortex is one of the most frequently reported findings in ASD [46]. Heightened neural excitability, however, is not likely sensory modality specific, as a recent auditory MEG study showed that ASD participants have increased neural responses to repeated stimuli while control participants show habituation of response over repetition [47]. Thus, our findings support a growing literature in indicating more general dysregulation of neural activity in sensory systems in ASD.

We observed a fair amount of heterogeneity in the ASD group with regard to neural excitability as suggested by slopes of the stimulus-response function with increasing stimulus contrast. The excitability to sensory stimulation was enhanced above the normal range in approximately 45 % of our ASD sample who showed greater response increases than any of TD participants. The remaining ASD participants had sensory responses that overlapped with TD participants. Thus, while our sample is not large, it appears that the enhanced excitability may be present in a substantial portion of our participants on the ASD spectrum but not all. This observation is consistent with a recent study suggesting that GABA-related neural inhibition may be reduced in a specific subgroup in ASD [48]. Thus, elevation in cortical excitability may represent a neurophysiological biomarker for parsing clinically meaningful heterogeneity in the ASD population.

Limitations of this study include the small sample size. Additionally, we did not find a significant correlation between the electrophysiological measures of cortical excitability and ASD severity, and thus, it is possible that the findings relate more specifically to sensory disturbances than broader clinical symptoms in ASD. Our data cannot discriminate between a deficit of reduced inhibitory tone and one of increased excitatory tone because EEG recording represents the net population neural activity influenced by both factors. Thus, questions about whether heightened neural excitability result from alterations of local inhibitory or excitatory circuit alterations in sensory cortex, heightened thalamic drive, or reduced inhibitory modulation by association cortex remain important questions for future research.

## Conclusions

We demonstrated that participants with ASD have greater neural excitability during sensory stimulation. We also showed that the heightened excitability appears to affect only a portion of individuals in this population and that it is highly associated with parental report of sensory processing symptoms that affect these individuals’ daily functioning. While pathophysiological mechanisms remain to be determined, our observations are promising from the perspective of identifying individuals who are most likely to benefit from treatments targeting this neurobiological abnormality and tracking their treatment outcomes. Further investigations are needed to better clarify the relevant biochemical and neurophysiological basis for the observed electrophysiological characteristic of sensory processing and its relationship to broader social and communication impairments in ASD.
